# Discrimination within Recognition Memory in Schizophrenia

**DOI:** 10.3390/bs3020273

**Published:** 2013-06-07

**Authors:** Kathryn A. McGuire, Melanie M. Blahnik, Scott R. Sponheim

**Affiliations:** 1 Minneapolis Veterans Affairs Health Care System, One Veterans Drive, Minneapolis, MN 55417, USA; E-Mails: Melanie.Blahnik@va.gov (M.M.B.); sponh001@umn.edu (S.R.S.); 2 Department of Psychiatry, University of Minnesota, F282/2A West, 2450 Riverside Avenue, Minneapolis, MN 55454, USA

**Keywords:** schizophrenia, recognition memory, verbal memory, nonverbal memory

## Abstract

Episodic memory is one of the most affected cognitive domains in schizophrenia. First-degree biological relatives of individuals with schizophrenia also have been found to exhibit a similar, but milder, episodic memory deficit. Unlike most studies that focus on the percent of previously presented items recognized, the current investigation sought to further elucidate the nature of memory dysfunction associated with schizophrenia by examining the discrimination of old and new material during recognition (measured by d') to consider false recognition of new items. Using the Recurring Figures Test and the California Verbal Learning Test (CVLT), we studied a sample of schizophrenia probands and the first-degree biological relatives of patients with schizophrenia, as well as probands with bipolar disorder and first-degree biological relatives to assess the specificity of recognition memory dysfunction to schizophrenia. The schizophrenia sample had poorer recognition discrimination in both nonverbal and verbal modalities; no such deficits were identified in first-degree biological relatives or bipolar disorder probands. Discrimination in schizophrenia and bipolar probands failed to benefit from the geometric structure in the designs in the manner that controls did on the nonverbal test. Females performed better than males in recognition of geometric designs. Episodic memory dysfunction in schizophrenia is present for a variety of stimulus domains and reflects poor use of item content to increase discrimination of old and new items.

## 1. Introduction

It has been well-established in the literature that neurocognitive dysfunction is associated with schizophrenia, a focus with historical roots dating back to the Kraepelinian era of “dementia praecox” (a term Emil Kraepelin derived from a Latin translation of Benedict Morel’s French term “demence precoce”) [1]. Although contemporary research has refuted the notion that schizophrenia involves an inevitable deteriorating course [2], Kraepelin’s nosology is remarkable for an astute recognition of the significance of the cognitive deficits associated with the disorder, as well as the postulated underlying organic brain dysfunction. Today, there is much evidence that neuropsychological deficits are a sensitive indicator of brain dysfunction and have been shown to be associated with genetic vulnerability for the disorder [3,4,5,6,7,8,9]. More specifically, research findings suggest that neuropsychological deficits are associated with genetically influenced brain structure and function that predisposes individuals to psychosis [10,11,12,13,14]. This area of study is also immediately relevant to the daily lives of people with schizophrenia, as cognitive functioning has been shown to be a reliable predictor of community functioning [15] and knowledge of deficient cognitive processes may lead to more effective psychosocial interventions and cognitive remediation for the disorder. 

Recent research efforts have also been committed to the identification and measurement of cognitive deficits in non-psychotic, first-degree biological relatives of individuals with schizophrenia. A number of studies have shown that first-degree biological relatives exhibit cognitive deficits that parallel those found in schizophrenia, but are less severe [16,17,18]. It has been proposed that these cognitive deficits are endophenotypic markers that may indicate increased susceptibility or genetic predisposition to developing the disorder [19,20,21,22]. It is thought that these markers are likely to be more closely related to the mechanism of gene action than the more conspicuous clinical phenotype [23,24]. Neuropsychological studies of relatives are valuable, because they may lead to a greater understanding of the disease processes associated with schizophrenia. More specifically, such studies may lead to identification of phenotypes that are more effective for genetic analysis, as well as obtaining a measure of cognitive functioning not confounded by neuroleptic treatment, chronic hospitalization and the potential neurotoxic effects of psychosis [25].

Considerable research efforts have been devoted to characterizing the cognitive deficits associated with schizophrenia over the past few decades, though disagreement remains concerning the precise nature of the cognitive impairments. There is some consensus that episodic memory is one of the most severely affected cognitive domains in schizophrenia [26,27,28,29,30,31,32,33]. In a meta-analysis, Aleman *et al.* [26] found that the memory deficits persisted across domains of memory (e.g., immediate, delayed and recognition), as well as stimulus (verbal *vs.* visual/nonverbal), although in a quantitative review, Heinrichs and Zakzanis [29] noted that nonverbal memory deficits demonstrate a more variable pattern across studies, which also has been confirmed in more recent studies [34]. Memory deficits, in particular, have been frequently identified in first-degree biological relatives of individuals with schizophrenia [4,8,23,25,35,36,37,38,39,40,41,42,43]. Some have hypothesized that the memory deficits observed in schizophrenia reflect a failure to utilize strategies to assist with recall, such as organizing or structuring material to be learned [44,45,46]. Complicating matters, there is further evidence of gender differences in performance in memory, as well as other cognitive domains, with female patients with schizophrenia generally demonstrating milder impairments than their male counterparts [47,48,49].

Evidence suggests that the memory deficits associated with schizophrenia are not simply an artifact of more global impairment or intelligence quotient (IQ) [29]. Heinrichs and Zakzanis [29] noted that, given the typical onset of this disorder in the late teenage years or early adulthood and, thus, the likelihood of the illness undermining academic success, measures of educational attainment or intellectual abilities may underestimate overall abilities. In addition, the cognitive impairments in schizophrenia have been found to be consistently more severe than those present in patients with affective disorders [26,50,51], thus suggesting that the deficits and, in particular, the degree of impairment may be specific to schizophrenia [52]. Finally, the literature has supported the notion that the cognitive deficits associated with schizophrenia are not accounted for by clinical symptomatology, such as psychosis [53], and are stable characteristics of individuals affected by the disorder or who carry substantial genetic liability for the condition. Specifically, cognitive deficits have been found prior to the onset of other clinical symptoms [31,54,55,56,57]; cognitive impairments also have been observed in relatives of schizophrenia patients who do not demonstrate other clinical symptoms [58], and the cognitive impairments have been shown to persist even during remission of clinical symptoms [59,60,61].

Despite consistent findings across studies of significant deficits in immediate and delayed free recall in individuals with schizophrenia, more variable findings have been observed in performance on tasks of recognition memory [62]. Recently, impaired performance has been observed in individuals with schizophrenia on recognition memory tasks that require discrimination between old and new items and appears to be associated with decreased activation in the fronto-parietal regions [63,64]. Given these findings, the role of discrimination within recognition memory in schizophrenia warrants further exploration. The overall objective of the current study was to further elucidate the nature of memory dysfunction associated with schizophrenia by examining both nonverbal and verbal recognition memory functioning and, more specifically, discrimination of old and new material during recognition (measured by d'), in a sample of schizophrenia probands and the first-degree biological relatives of patients with schizophrenia. Specificity of memory dysfunction to schizophrenia, relative to other severe mental disorders, was further explored through comparisons with bipolar probands and first-degree biological relatives of patients with bipolar disorder. Hypotheses were as follows:

1) Probands with schizophrenia were expected to demonstrate significantly lower discrimination (d') on nonverbal and verbal tasks of recognition memory compared to nonpsychiatric controls and probands with bipolar disorder.
2) Relatives of patients with schizophrenia were expected to demonstrate significantly lower discrimination (d') compared to nonpsychiatric controls and relatives of patients with bipolar disorder, for both nonverbal and verbal material.
3) Recognition of geometric designs was expected to be higher than recognition of nonsense designs across all groups. However, probands with schizophrenia and relatives of patients with schizophrenia were expected to demonstrate a significantly lower benefit from geometric design types compared to nonpsychiatric controls, probands with bipolar disorder and relatives of patients with bipolar disorder.
4) In comparison to controls and probands with bipolar disorder, probands with schizophrenia were expected to demonstrate significantly greater use of a serial strategy (a less efficient strategy) and significantly lower use of a semantic strategy for recall of a list of words. A similar pattern was expected in first-degree biological relatives of patients with schizophrenia.

Given findings in the literature regarding gender differences in performance, gender was added as an additional factor in the above analyses. Female probands with schizophrenia were expected to perform better than their male counterparts across discrimination variables. Supplemental analyses also were run without gender as a factor and have been summarized in a separate section. 

## 2. Method

### 2.1. Participants

Forty individuals with schizophrenia, 30 individuals with bipolar disorder and 65 nonpsychiatric controls of a similar age participated in the study. In addition, 52 first-degree biological relatives of individuals with schizophrenia and 31 first-degree relatives of individuals with bipolar disorder were included for comparison. 

#### 2.1.1. Patient Probands

Individuals with schizophrenia and bipolar disorder were recruited from outpatient clinics at the Minneapolis Veterans Affairs Health Care System or from community and county mental health clinics. Diagnoses were determined using the Diagnostic Interview for Genetic Studies (DIGS) [65], administered by a doctoral-level clinician. Participants further provided consent for a review of their available medical records, which provided corroboration of information obtained during the interview, including medications. Diagnoses that differed between the interviewer and reviewer were carefully reviewed and discussed to attain consensus. The primary interviewer also assessed participants’ positive, negative and affective symptomatology utilizing the Scale for the Assessment of Positive Symptoms (SAPS) [66], the Scale for the Assessment of Negative Symptoms (SANS) [67] and the Brief Psychiatric Rating Scale (BPRS, 24-item version) [68], based on impressions from the clinical interview. For those probands prescribed medication, dosage was estimated using chlorpromazine (CPZ) equivalent measures. Probands were included in the study if they met DSM-IV [69] criteria for any subtype of schizophrenia or bipolar I disorder. All participants were outpatients with minimal symptomatology based on mean scores on the SAPS, SANS and BPRS that fell predominantly in the very mild to mild range (see Table 1). Potential participants were excluded if they had English as a second language, a charted or reported IQ of less than 70 or a diagnosis of mental retardation, current alcohol or drug abuse, past drug dependence, a current or past central nervous system disease or condition, a medical condition or disease with likely significant central nervous system effects, history of head injury with skull fracture or loss of consciousness of greater than 20 min, a physical problem that would render study measures difficult or impossible to administer or interpret (blindness, hearing impairment, paralysis in the upper extremities), age less than 20 or greater than 59, significant tardive dyskinesia, as indicated by a Dyskinesia Identification System: Condensed User Scale (DISCUS) [70], a history of electro-convulsive therapy or a history of having been adopted. 

**Table 1:** Participant characteristics: demographic and clinical information for schizophrenia probands, bipolar probands and nonpsychiatric controls.

Variable	Schizophrenia probands	Bipolar probands	Controls	Test value (df)	p-value
	(N = 40)	(N = 30)	(N = 65)		
Age (Years)	44.75 (10.2)	44.03 (9.8)	44.57 (13.5)	*F*(2,132) = 0.03	n.s. ^a^
Gender (M/F)	33/7	23/7	29/36	χ^2 ^(2,N = 145) = 18.4	< 0.001
Ethnicity					
Caucasian	35/37	29/29	56/57	NA	NA
African American	2/1	0/0	5/5	NA	NA
Hispanic	1/0	0/0	0/0	NA	NA
Asian	1/1	0/0	0/0	NA	NA
Native American	1/1	1/1	2/1	NA	NA
#x2003;Other	0/0	0/0	2/2	NA	NA
Education (Years)	13.95 (2.3)	15.17 (2.1)	15.11 (1.9)	*F*(2,132) = 4.61	0.012 ^a^
Estimated IQ	96.75 (12.9)	111.90 (16.1)	109.71 (11.0)	*F*(2,132) = 16.19	< 0.001 ^a^
SAPS Mean Score	0.54 (0.5)	0.18 (0.2)	NA	*t*(57.24) = 4.30	< 0.001 ^b,c^
SANS Mean Score	0.91 (0.5)	0.33 (0.3)	NA	*t*(61.57) = 6.49	< 0.001 ^b,c^
BPRS Mean Score	1.79 (0.5)^N = 39^	1.49 (0.4)^N = 29^	NA	*t*(65.99) = 2.80	0.007 ^b,c^
CPZ (mg)	646 (442)^N = 14^	316.67 (76.4)^N = 3^	NA	*t*(14.98) = 2.61	0.020 ^b,c^

Notes: Numbers represent group means and standard deviations (in parentheses) for all variables, except gender and ethnicity, where numbers represent total count. n.s. = not significant. NA = not applicable. IQ = Intelligence Quotient. Estimated IQ was derived from the formula of Brooker and Cyr [71] using the Vocabulary and Block Design subtests. SAPS = Scale for the Assessment of Positive Symptoms (34 items); range = 0–5 (absent to severe). SANS = Scale for the Assessment of Negative Symptoms (25 items); range = 0–5 (absent to severe). BPRS = Brief Psychiatric Rating Scale, 24-item version; range = 1–7 (absent to extremely severe). ^a^ Denotes significance level for a one-way ANOVA. ^b^ Denotes significance level for an independent samples *t*-test. ^c^ Denotes significance level when equal variance is not assumed.

#### 2.1.2. First-Degree Biological Relatives

First-degree relatives were recruited using contact information provided by the proband participants, followed by a telephone interview. In an effort to optimize participation, relatives were excluded only if they had physical conditions that precluded them from being able to participate in the study protocol or if they were younger than 20 or older than 70. Schizotypal signs were evaluated using the Schizotypal Personality Questionnaire (SPQ) [72]. In addition, to assess for DSM-IV Axis I and II symptomatology, including schizotypy, structured interviews were conducted, including the Structured Clinical Interview for DSM-IV (SCID) [73], Structured Clinical Interview for DSM-IV Personality Disorders (SCID-II) [74] and Structured Interview for Schizotypy (SIS) [75].

Probands contributed different numbers of family members to the study. The variance attributable to family membership is only a possible influence in the analysis of relatives, since individuals in the proband and control samples were unrelated. The relatives in the study came from 41 families, with an average of 1.85 individuals enrolled from each family. For families affected by schizophrenia, an average of 1.87 individuals participated, and for families affected by bipolar disorder, an average of 1.82 individuals participated. To address the possible confound of family membership in the analysis of relatives, we carried out multi-level analyses that included family membership as a dependent variable. Results showed that family membership failed to account for a significant portion of the variance in any dependent variable. Intra-class correlations (ICC), summarizing the amount of variance attributable to family membership, were nonsignificant and minimal for all dependent variables (Recurring Figures Test ICC’s: all indices, *r* = 0.00; CVLT: d' recognition, *r* = 0.09, semantic clustering index, *r* = 0.13, serial clustering index, *r* = 0.15). Because there were no group differences for the relatives in any recognition memory variable and the variables have nonsignificant and negligible amounts of variance related to family membership, findings are essentially unaffected by this factor.

#### 2.1.3. Nonpsychiatric Controls

Nonpsychiatric controls were recruited through postings at community libraries, fitness centers and the Minneapolis Veterans Affairs Health Care System, as well as from advertisements in veteran and other organization newsletters. They were screened for the same exclusion criteria as the schizophrenia probands, with the exception of age, which ranged from 20 to 70. Additionally, an interview of medical and personal history was conducted to rule out a personal history of psychotic or affective disorder or a first-degree biological relative with a history of psychotic or affective disorder requiring hospitalization or medication. Finally, the SCID, SCID-II and SIS were completed to assess DSM-IV Axis I and Axis II symptomatology. Table 1 presents comparisons of characteristics between the probands and nonpsychiatric controls in the study. Table 2 comprises comparisons of characteristics between the first-degree biological relatives and nonpsychiatric controls.

**Table 2:** Participant characteristics: demographic and clinical information for first-degree biological relatives of schizophrenia patients, first-degree biological relatives of bipolar patients and nonpsychiatric controls.

Variable	Schizophrenia relatives	Bipolar relatives	Controls	Test value (df)	*p*-value
	(N = 52)	(N = 31)	(N = 65)		
Age (Years)	49.85 (11.1)	46.45 (14.3)	44.57 (13.5)	*F*(2,145) = 2.45	n.s. ^a^
Gender (M/F)	20/32	19/12	29/36	χ^2^(2,N = 148) = 4.2	n.s.
Ethnicity					
Caucasian	46/47	30/30	56/57	NA	NA
African American	2/2	0/0	5/5	NA	NA
Hispanic	4/1	0/0	0/0	NA	NA
Asian	0/2	0/0	0/0	NA	NA
Native American	0/0	0/1	2/1	NA	NA
Other	0/0	1/0	2/2	NA	NA
Education (Years)	14.62 (2.3)	14.32 (3.1)	15.11 (1.9)	*F*(2, 145) = 1.37	n.s. ^a^
Estimated IQ	105.17 (14.0)	108.97 (15.3)	109.71 (11.0)	*F*(2, 145) = 1.85	n.s. ^a^
SPQ Total Score	14.85 (10.0)^N = 48^	16.39 (15.1)^N = 28^	10.38 (6.2)^N = 64^	*F*(2, 137) = 4.76	0.010 ^a^

Notes: Numbers represent group means and standard deviations (in parentheses) for all variables except gender and ethnicity, where numbers represent total count. n.s. = not significant. NA = not applicable. IQ = Intelligence Quotient. Estimated IQ was derived from the formula of Brooker and Cyr [71] using the Vocabulary and Block Design subtests. SPQ = Schizotypal Personality Questionnaire. ^a^ Denotes significance level for a one-way ANOVA.

### 2.2. Procedures

The California Verbal Learning Test (CVLT) [76] and a computerized version of the Recurring Figures Test [77] were administered as part of a larger cognitive battery. In addition, participants completed the Vocabulary and Block Design subsets of the Wechsler Adult Intelligence Scale—Third Edition (WAIS-III), and an estimated Intelligence Quotient (IQ) was derived using a formula from Brooker and Cyr [71]. A subgroup of participants who had completed the entire Recurring Figures Test was selected from the larger study sample. These individuals also had completed the CVLT, with the exception of one individual with bipolar disorder.

#### 2.2.1. Recurring Figures Test

The Recurring Figures Test consists of 160 cards, 80 of which portray a geometric design, while the other 80 portray an irregular nonsense design. Individuals are exposed to the cards for three seconds at a time over the course of 8 trials (20 cards per trial). Eight cards, including 4 geometric designs and 4 nonsense designs, are repeated in all eight trials, with all other cards exposed only once. Following exposure to the first trial, examinees are instructed to indicate in the remaining trials which designs they have seen in prior trials. The last two trials (Trials 7 and 8) occur after a delay of 45 to 60 min, allowing for measurement of delayed recognition.

For the present study, an experimental computerized version of the Recurring Figures Test was created with the permission of the original author (Dr. Doreen Kimura). Scanned versions of the original cards were flashed in front of the participants for 3 s. Following the first trial of 20 cards, participants were asked to press one button if they had seen the design previously and a different button if they had not previously seen the design. All 160 cards were administered, across eight trials, including Trials 7 and 8, following a delay of 45 min from the completion of Trial 6. During the course of the development of the program, an error occurred in one of the 8 key cards for Trial 8, resulting in 5 nonsense key designs (one nonsense figure appearing twice) and 3 geometric key designs being shown in this trial. d' calculations were adjusted accordingly.

#### 2.2.2. California Verbal Learning Test (CVLT)

The CVLT is a list learning task, which involves the oral presentation of a list of 16 words (List A) over five immediate recall trials, such that the subject is required to recall from memory as many of the words as they can after each presentation of the list. The list consists of four words from each of four categories: fruits, clothing, tools and herbs and spices. Adjacent words on the list are from different categories, allowing the examiner to determine the degree to which the participant utilizes semantic organization strategies to encode the words. Following the five trials, an interference list (List B) is presented for one trial, after which free recall of List A is again tested (short delay free recall), followed by category-cued recall of List A (short delay cued recall). Recall is tested again after a 20-min interval (during which nonverbal testing occurs) in a free recall format (long delay free recall), followed by a category cued recall format (long delay cued recall). Recognition testing then occurs in a yes-no format: each word from a list of 44 words is orally presented one at time, after which the subject responds with yes if it was on List A and no if it was not. The 44-item recognition list consists of the 16 List A items interspersed with 28 distracters (List B words semantically related to List A words, List B words semantically unrelated to List A words, novel words prototypical of semantic categories represented in List A, novel words phonetically similar to List A words and novel words semantically and phonetically unrelated to List A words). The format of the test permits assessment of multiple aspects of learning and memory, including overall recall ability, rate of learning over repeated trials, learning strategy (e.g., semantic clustering or serial memorization), ability to retain learned material over time and recognition memory. In the present study, discrimination within recognition memory was the primary measure of interest and, thus, measures of free recall were not examined, with the exception of comparing serial and semantic strategy to determine whether semantic structure in verbal material showed effects similar to geometric structure in figural material.

### 2.3. Measure of Discrimination in Recognition Memory

As the Recurring Figures Test is a visual recognition task, the ability to discriminate between new and repeated materials during recognition phases (*i.e*., discrimination index) was the primary variable of interest. To evaluate discrimination, d' was calculated for both the Recurring Figures Test and the CVLT using the following formula [78,79]:

d' = Z(HR) − (FAR)(1)

where Z = Z-score, HR = hit rate (*i.e*., number of correct “yes” responses) and FAR = false alarm rate (*i.e*., proportion of “no” trials to which the participant responded “yes”). For the Recurring Figures Test, separate d´ variables were calculated for Trials 2 through 6 (immediate recognition) and Trials 7 through 8 (delayed recognition). Separate d´ scores also were calculated for geometric and nonsense designs for both immediate and delayed recognition. For the CVLT, d' could only be calculated for the recognition condition of the task, due to the unavailability of false negatives and true negatives in the free recall portion of the test. 

#### 2.3.1. Recurring Figures Test

For d**'**, repeated-measures analysis of variance (ANOVA) was used, with group (schizophrenia, bipolar disorder and nonpsychiatric controls or relatives of patients with schizophrenia, relatives of patients with bipolar disorder and nonpsychiatric controls) and gender (male *vs.* female) as between-subjects factors and time delay (immediate *vs.* delayed) as the within-subjects factors. Follow-up analyses for significant findings included repeated-measures one-way ANOVA for group effects, followed by *post hoc* Tukey’s HSD test and paired *t*-tests for time delay effects. Analyses also were run using stimulus (geometric *vs.* nonsense) as an additional within-subjects factor to explore any differences in the ease of recognition of structured *vs.* unstructured nonverbal information.

#### 2.3.2. CVLT

For d**'**, repeated-measures ANOVA also was used to assess group and gender differences for the CVLT recognition condition. Follow-up analyses for significant effects included, as with the Recurring Figures Test, one-way ANOVA for significant group effects and, when indicated, *post hoc* Tukey's HSD test. To further assess for differences in use of semantic information used to assist with episodic memory, semantic *vs.* serial recall style was assessed using Semantic Cluster Ratio and Serial Cluster Ratio scores obtained from the scoring printout of the CVLT (The Psychological Corporation, 1987). A repeated-measures ANOVA was run with group (schizophrenia, bipolar disorder and nonpsychiatric controls or relatives of patients with schizophrenia, relatives of patients with bipolar disorder and nonpsychiatric controls) and gender (male *vs.* female) as between-subjects factors and recall style (semantic *vs.* serial) as a within-subjects factor, with follow-up one-way ANOVA and *post hoc* Tukey’s HSD tests, as indicated.

## 3. Results

Comparisons of discrimination between old and new items were run separately for probands (schizophrenia *vs.* bipolar disorder) and first-degree biological relatives, using the same control group in each comparison. The comparison of patients allowed examination of the diagnostic specificity of recognition memory discrimination deficits, while the contrast of relatives tested whether any recognition abnormalities were specific to the genetic liability for schizophrenia. Mean d´ scores for the Recurring Figures Test and the CVLT are summarized in Table 3.

**Table 3:** Mean d´ scores (and standard deviation) for the Recurring Figures Test and CVLT.

Variable	Schizophrenia probands	Bipolar probands	Controls	Schizophrenia relatives	Bipolar relatives
	(N = 40)	(N = 30)	(N = 65)	(N = 52)	(N = 31)
RFT: d' Total	1.53 (0.76) ^a,c^	2.05 (0.91) ^c^	2.16 (0.71) ^a^	2.18 (0.65)	2.16 (0.55)
(Trials 2–6)					
RFT: d' Total	1.48 (0.93) ^a,b^	2.24 (1.10) ^b^	2.39 (0.76) ^a^	2.19 (0.88)	2.20 (0.74)
(Trials 7–8)					
RFT: d' Geometric	2.53 (1.16) ^a,c^	3.17 (1.21)^c^	3.45 (0.95) ^a^	3.55 (0.87)	3.33 (0.88)
Designs (Trials 2–6)					
RFT: d' Geometric	2.82 (1.43) ^a^	3.24 (1.31)	3.83 (0.95) ^a^	3.70 (0.90)	3.71 (0.99)
Designs (Trials 7–8)					
RFT: d' Nonsense	1.05 (0.73) ^b,c^	1.54 (0.94)^c^	1.59 (0.82) ^b^	1.55 (0.73)	1.58 (0.75)
Designs (Trials 2–6)					
RFT: d' Nonsense	0.92 (1.03) ^a,b^	1.89 (1.17)^b^	1.79 (1.07) ^a^	1.62 (1.26)	1.47 (1.19)
Designs (Trials 7–8)					
CVLT: d' Recognition	2.63 (0.99) ^b1,b2^	3.61 (0.86)^b1,N = 29^	3.54 (0.86) ^b2^	3.42 (0.93)	3.64 (0.79)

Notes: CVLT = California Verbal Learning Test. d´ = D-prime. RFT = Recurring Figures Test. ^a^ Denotes a significant difference in paired comparisons (Tukey's HSD) at the *p* < 0.001 level. ^b, b1, b2^ Denote a significant difference in paired comparisons (Tukey's HSD) at the *p* < 0.01 level. ^c^ Denotes a significant difference in paired comparisons (Tukey's HSD) at the *p* < 0.05 level.

### 3.1. Recurring Figures Test

**Figure 1:**
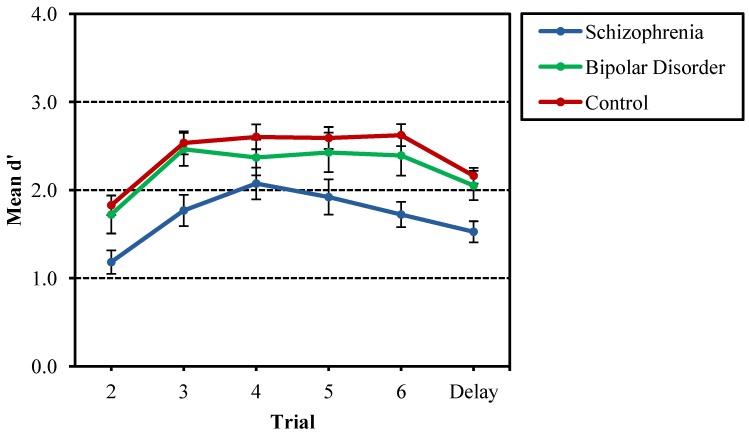
Mean d' across Trials 2–6 and delayed trials (7–8) for the Recurring Figures Test for probands with schizophrenia and bipolar disorder, as well as nonpsychiatric controls. Schizophrenia probands were significantly lower in recognition discrimination (d**'**) compared to bipolar disorder and control probands, and performance on Trial 2 was significantly lower than other trials. There was no interaction of group and trial number.

Comparisons of d´ between proband groups and nonpsychiatric controls on the Recurring Figures Test resulted in significant effects for time delay, *F*(1, 129) = 8.32, *p* = 0.005, and group, *F*(2, 129) = 6.11, *p* = 0.003. Follow-up analyses for both the immediate (Trials 2–6) and delayed (Trials 7–8) conditions yielded group effects for both immediate recognition, *F*(2, 132) = 5.21, *p* < 0.001, and delayed recognition, *F*(2, 132) = 13.44, *p* < 0.001. In both conditions, probands with schizophrenia obtained significantly lower d´ scores than nonpsychiatric controls and probands with bipolar disorder, while the bipolar disorder probands did not differ from controls. For the time delay main effect, paired *t*-tests revealed that d´ for the delayed recognition condition for all participants was higher than d´ for the immediate recognition condition, *t*(134) = 2.53, *p* = 0.012. This difference in d´ scores between immediate and delayed conditions likely reflects the benefits of increased exposure to the eight stimulus designs across eight trials (e.g., earlier trials with lower d´ scores pulled down the average mean for the immediate recognition condition). To explore this finding, d´ was calculated individually for Trials 2 through 6. Figure 1 shows the mean d´ growth curve for Trials 2–6 and the delayed trials (7–8). Paired samples *t*-tests comparing mean d´ for Trials 2–6 with groups collapsed, demonstrating that mean d´ for Trial 2 was lower than that of Trials 3–6, with *p* < 0.001 across all comparisons; Trials 3–6 did not differ from each other. Repeated-measures ANOVA did not yield significant interaction between group and trial, suggesting similar growth curves across the three groups.

**Figure 2:**
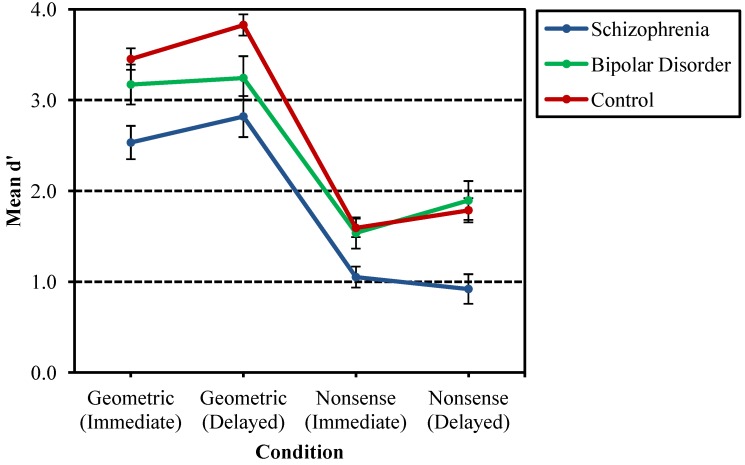
Comparison of mean d' for schizophrenia probands, bipolar probands and nonpsychiatric controls by stimulus and time delay. Probands with schizophrenia had lower d´ scores compared to both nonpsychiatric controls and probands with bipolar disorder in all conditions, except delayed recognition for geometric designs, where bipolar probands did not differ significantly from either the schizophrenia probands or the controls.

With respect to d**' **scores calculated separately for geometric and nonsense designs, main effects were found for stimulus, *F*(1, 129) = 263.47, *p* < 0.001, time delay, *F*(1, 129) = 15.96, *p* < 0.001, group, *F*(2, 129) = 6.80, *p* = 0.002, a two-way interaction of stimulus and group, *F*(2,129) = 3.08, *p* = 0.049, and three-way interactions of stimulus, group and gender, *F*(2, 129) = 3.54, *p* = 0.032, as well as stimulus, time delay and group, *F*(2, 129) = 3.16, *p* = 0.046. The two-way interaction of stimulus and time delay also approached significance, *F*(1, 129) = 3.41, *p* = 0.067. Follow-up comparisons for each of the four conditions ((1) geometric designs—immediate recognition; (2) geometric designs—delayed recognition; (3) nonsense designs—immediate recognition; (4) nonsense designs—delayed recognition) showed group effects in each condition ((1) geometric designs—immediate recognition: *F*(2, 132) = 9.13, *p* < 0.001; (2) geometric designs—delayed recognition: *F*(2, 132) = 9.12, *p* < 0.001; (3) nonsense designs—immediate recognition: *F*(2, 132) = 5.74, *p* = 0.004; (4) nonsense designs—delayed recognition: *F*(2, 132) = 9.88, *p* < 0.001). *Post hoc* analysis revealed probands with schizophrenia to have lower d' scores compared to both nonpsychiatric controls and probands with bipolar disorder in all conditions, except delayed recognition for geometric designs, where bipolar probands did not differ significantly from either the schizophrenia probands or the controls. Schizophrenia probands did have lower d´ scores than controls. Figure 2 depicts the interactions between stimulus, time delay and group. 

**Figure 3:**
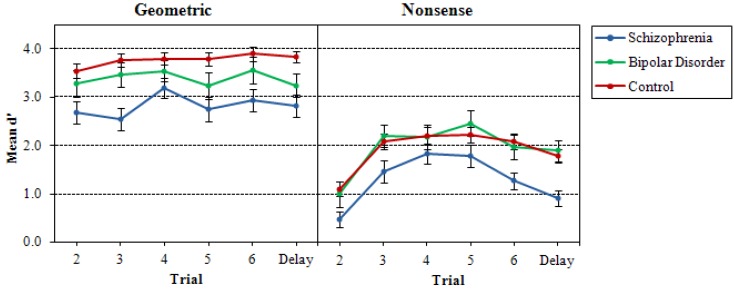
Mean d' for geometric and nonsense designs across Trials 2–6 and delayed trials (7–8) for probands with schizophrenia and bipolar disorder and nonpsychiatric controls.

In follow-up analyses of the time delay effects, paired *t*-tests of immediate and delayed conditions for geometric and nonsense designs revealed higher d' scores for the delayed trials compared to the immediate trials for geometric designs only, *t*(134) = 3.92, *p* < 0.001. Figure 3 shows the differences in d' growth curves for the three groups between geometric and nonsense designs.

As can be seen by the graphs, growth curves across trials were flatter for geometric designs than for nonsense designs. The significant stimulus effect also was explored with paired samples *t*-tests, which demonstrated the expected finding that mean d' for the nonsense figures was lower than mean d´ for geometric figures for both immediate recognition, *t*(134) = 18.74, *p* < 0.001, and delayed recognition, *t*(134) = 16.01, *p* < 0.001.

**Figure 4:**
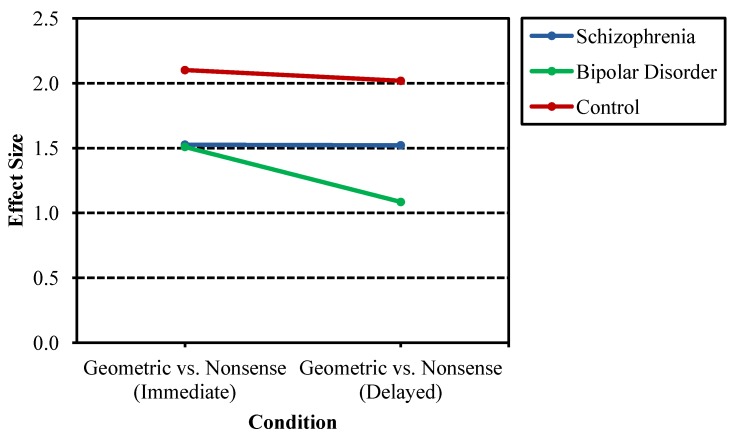
Effect sizes of d' differences between geometric designs and nonsense designs across proband groups and nonpsychiatric controls. Schizophrenia probands showed less benefit than psychiatric controls from namable geometric structures regardless of condition. Bipolar probands also showed less benefit from namable geometric structures in both conditions, but additionally demonstrated greater loss in benefit after a delay as compared to the other groups.

Additional follow-up analyses to the stimulus-by-group and stimulus-by-time delay-by-group interactions allowed examination of the benefits of more easily namable figures (e.g., triangle) as compared to nonsense figures akin to scribbles. Effect sizes also were calculated, reflecting the difference in mean d´ between the two design types for each group divided by the pooled standard deviation for each group (Figure 4). The effect size for schizophrenia probands is similarly below that of controls for both immediate and delayed recognition conditions, suggesting less improvement with the presence of geometric structure regardless of condition. Bipolar probands, while also demonstrating less improvement with the presence of geometric structure compared to nonpsychiatric controls, further demonstrated a greater decline in effect size between immediate and delayed recognition relative to schizophrenia probands and nonpsychiatric controls. This finding is consistent with the bipolar probands benefiting less from geometric structure in the design after a delay as compared to the other groups. 

Findings related to the gender effect in the probands should be interpreted with caution, since there were only seven females in each patient group. Females with schizophrenia demonstrated a greater mean d' difference (pooled across immediate and delayed conditions) between geometric and nonsense figures than males with schizophrenia, *t*(38) = 2.46, *p* = 0.019, whereas no significant differences were seen between gender groups for probands with bipolar disorder and nonpsychiatric controls. The benefit from nameable geometric structures that appeared greater for females with schizophrenia than males with the disorder suggests that they are better able to compensate for episodic memory deficits by using semantic strategies in encoding and retrieval. *t*-tests of d' scores within groups revealed that female probands with schizophrenia performed significantly better than male probands with schizophrenia on delayed recognition of geometric designs, *t*(38) = 2.06, *p* = 0.046, while performing similarly to their male counterparts in the three other conditions. No significant gender differences were found in any of the four conditions for bipolar probands and nonpsychiatric controls. Again, the effect of gender in schizophrenia probands should be considered preliminary, due to the limited number of female probands.

**Figure 5:**
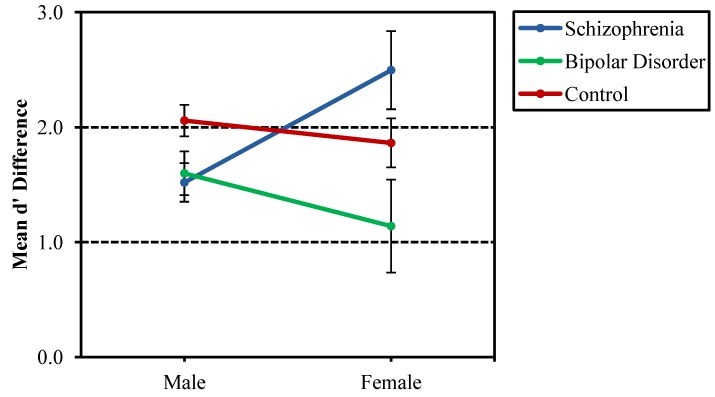
Mean d' difference scores (nonsense d' subtracted from geometric d') separated by group (probands with schizophrenia, probands with bipolar disorder and nonpsychiatric controls) and gender. Females with schizophrenia appeared to benefit more greatly from nameable geometric structure in figures as compared to males with schizophrenia, but this difference should be interpreted with caution, since there were only seven females in each of the schizophrenia and bipolar disorder probands groups.

In comparing first-degree biological relatives of patients with schizophrenia and bipolar disorder to nonpsychiatric controls, no significant effects were found for d'. When d' scores were further broken down by geometric and nonsense designs, effects were found for stimulus, *F*(1, 142) = 433.27, *p* < 0.001, time delay, *F*(1, 142) = 11.57, *p* = 0.001, and stimulus-by-time delay, *F*(1, 142) = 5.68, *p* = 0.018. For the significant stimulus effect, follow-up paired samples *t*-tests were used comparing mean d´ for geometric and nonsense designs in the immediate and delayed recognition conditions. Differences were found in both conditions (immediate recognition: *t*(147) = 22.30, *p* < 0.001; delayed recognition: *t*(147) = 18.24, *p* < 0.001), with d´ for geometric designs significantly higher than d´ for nonsense designs. As with proband analyses, follow-up exploration of the time delay main effect revealed higher mean d' for delayed recognition of geometric designs than for immediate recognition of geometric designs, *t*(147) = 4.64, *p* < 0.001. For nonsense designs, there were no differences between mean d' for immediate *vs.* delayed recognition.

Within the group of first-degree biological relatives and psychiatric controls, a trend was seen for the delay and gender interaction in comparisons of both total d', *F*(1, 142) = 3.85, *p* = 0.052, and d' calculated separately for geometric and nonsense designs, *F*(1, 142) = 3.66, *p* = 0.058. Paired-sample *t*-tests of d' calculated separately for geometric and nonsense designs demonstrated lower mean d´ in immediate *vs.* delayed recognition of geometric designs for both males, *t*(67) = 4.28, *p* < 0.001, and females, *t*(79) = 2.33, *p* = 0.022. Similar analyses for nonsense designs did not demonstrate differences for either gender.

### 3.2. CVLT

Significant group, *F*(2, 128) = 6.69, *p* = 0.002, and gender, *F*(1, 128) = 5.70, *p* = 0.018, effects were seen for d´ contrasts between proband groups and nonpsychiatric controls on the recognition condition of the CVLT. *Post hoc* analysis revealed that probands with schizophrenia on average earned lower d' scores relative to controls and probands with bipolar disorder, who did not differ from each other. The main effect for gender reflected a higher performance in the females as compared to the males. When specific groups were examined, no significant gender difference was found within each group, likely reflecting the smaller sample sizes.

Analysis of d' in first-degree biological relatives of probands with schizophrenia and bipolar disorder demonstrated a significant effect for gender only, *F*(1, 142) = 4.10, *p* = 0.045. Female relatives of individuals with schizophrenia demonstrated a greater mean d' difference in the CVLT than male relatives of individuals with schizophrenia, *t*(50) = 2.22, *p* = 0.031, whereas no significant differences were seen between gender groups for relatives of individuals with bipolar disorder and nonpsychiatric controls.

Analysis of semantic *vs.* serial recall yielded no significant findings for comparisons between patient probands and nonpsychiatric controls. Comparisons between first-degree biological relatives and nonpsychiatric controls yielded trends for an interaction between recall style and group, *F*(2, 142) = 2.69, *p* = 0.071, and recall style and gender, *F*(1, 142) = 3.81, *p* = 0.053. Follow-up analysis for the recall style-by-group interaction did not yield significant results. Follow-up independent samples *t*-tests of the recall style-by-gender interaction showed that females were more likely than males to utilize semantic recall, *t*(146) = 2.28, *p* = 0.024. There were no gender differences in utilization of serial recall.

### 3.3. Correlation Analysis

Bivariate (Pearson) correlation analysis was used to explore associations between recognition discrimination performance (d') on both the Recurring Figures Test and the CVLT and IQ estimate in the pooled proband and control group, as well as in the pooled relatives and control group. In both instances, better recognition discrimination (e.g., higher d' scores) was positively associated with estimated IQ across all conditions of the Recurring Figures Test (immediate and delayed total, geometric and nonsense design recognition), as well as the CVLT. These findings are consistent with the expectation that higher general intelligence would contribute to better performance across most cognitive domains. Within the pooled proband/control group, correlation coefficients between d' and IQ estimate ranged from 0.46 (delayed recognition of geometric designs) to 0.58 (immediate recognition of all designs), with all *p*-values falling below the 0.001 level of significance. Within the pooled relatives/control group, correlation coefficients between d' and IQ estimate ranged from 0.27 (immediate and delayed recognition of geometric designs) to 0.34 (immediate and delayed recognition of all designs), with all *p*-values also falling at or below the 0.001 level of significance.

We also carried out bivariate correlations comparing d' for the CVLT to d' for each condition of the Recurring Figures Test. These analyses showed generally higher positive correlations in the probands with bipolar disorder and nonpsychiatric controls than in the probands with schizophrenia. Correlations between d' for the CVLT and d' for each Recurring Figures Test condition in probands with schizophrenia ranged from 0.18 (immediate recognition of geometric designs) to 0.37 (delayed recognition for all designs), with most correlations falling in the 0.2 range. Only one correlation coefficient was significant at the 0.05 level, and no correlations were significant at the 0.01 level. For probands with bipolar disorder, correlation coefficients ranged from 0.22 (immediate recognition of geometric designs) to 0.50 (delayed recognition of nonsense designs). One of six correlations was significant at the 0.01 level, and four correlations were significant at the 0.05 level. Nonpsychiatric controls demonstrated correlation coefficients ranging from 0.20 (delayed recognition of nonsense designs) to 0.46 (immediate recognition for all designs). Five of six correlations were significant at the 0.01 level, while the sixth correlation was non-significant. Overall, these findings suggest lower associations between verbal and visual discrimination in recognition memory in individuals with schizophrenia relative to nonpsychiatric controls and individuals with bipolar disorder. This may be consistent with select disruptions in visual recognition memory functions being specific to schizophrenia patients. The generally modest correlations between Recurring Figures Test and CVLT indices demonstrate that most of the variance in performance on each task is unshared with the other domain and, thus, points to the verbal and visual tasks capturing unique variability in recognition memory.

Within the schizophrenia and bipolar proband group, higher medication dosage (as assessed by CPZ equivalent measures) predicted lower d' scores across all conditions of the Recurring Figures Test, as well as the CVLT, with Pearson correlation coefficients ranging from −0.19 (delayed recognition of nonsense designs, *p* = 0.031) to −0.37 (immediate recognition of geometric designs, *p* < 0.001). Likewise, higher ratings of both negative (SANS) and positive (SAPS) symptoms predicted poorer performance across conditions and modalities (nonverbal and verbal). Significant negative correlation coefficients in the 0.27 to 0.34 range (with *p*-values below the 0.05 or 0.01 level of significance) were observed between SANS total score and mean d´ for all variables except delayed recognition of nonsense designs. Significant negative correlation coefficients in the 0.24 to 0.30 range (*p* < 0.05) were observed between SAPS total score and mean d´ for delayed total recognition, delayed recognition of nonsense designs, immediate recognition of geometric designs and CVLT. Not surprisingly, higher ratings of negative and positive symptoms also were associated with higher medication dosage (n = 68, *r* = 0.32 and 0.31, respectively, *p* = < 0.01). Higher ratings of affective symptoms (BPRS total score) were not generally associated with performance; a small, but significant, positive association was seen between BPRS total score and CPZ equivalent measures (n = 66, *r* = 0.28, *p* = 0.023). Generally, the above findings suggest that for the proband group, greater severity of symptoms associated with schizophrenia, in particular, negative symptoms, is related to higher medication dosage and poorer nonverbal and verbal recognition discrimination. Within the relatives group, schizotypal signs (as measured by total SPQ score) failed to be associated with performance in nonverbal or verbal recognition discrimination.

### 3.4. Supplemental Analyses

As there were a limited number of female individuals in the proband groups, supplemental proband analyses were also carried out without gender as a factor. The removal of gender as a factor yielded similar results. On the Recurring Figures Test, comparisons of d' between proband groups and nonpsychiatric controls resulted in significant effects for time delay, *F*(1, 132) = 4.76, *p* = 0.031, and group, *F*(2, 132) = 12.85, *p* < 0.001. *Post hoc* analysis showed probands with schizophrenia to have lower d´ scores than nonpsychiatric controls and probands with bipolar disorder, while the bipolar disorder probands did not differ from controls. When stimulus type was added to the analyses, main effects were found for stimulus, *F*(1 132) = 337.96, *p* < 0.001, time delay, *F*(1, 132) = 11.93, *p* = 0.001, and group, *F*(2, 132) = 13.38, *p* < 0.001. *Post hoc* analysis again revealed lower d' scores in probands with schizophrenia compared to nonpsychiatric controls and probands with bipolar disorder, while the bipolar disorder probands did not differ from controls. The two-way interaction of stimulus and group, *F*(2, 132) = 2.21, *p* = 0.114, and the three-way interaction of stimulus, time delay and group, *F*(2, 132) = 2.97, *p* = 0.055, fell to a trend level. On the CVLT, d' comparisons without gender as a factor showed a main effect for group, *F*(2, 131) = 15.10, *p* < 0.001. *Post hoc* analysis revealed that probands with schizophrenia on average earned lower d´ scores relative to controls and probands with bipolar disorder, who did not differ from each other. Comparisons of semantic and serial recall again yielded no significant findings for comparisons between patient probands and nonpsychiatric controls.

## 4. Discussion

Consistent with hypotheses and prior research, probands with schizophrenia performed more poorly than nonpsychiatric controls and probands with bipolar disorder in both nonverbal and verbal recognition modalities. Likewise, as hypothesized, on the nonverbal memory task (Recurring Figures Test), schizophrenia probands demonstrated less benefit from the geometric design type across immediate and delayed conditions compared to nonpsychiatric controls, as evidenced by smaller effect sizes in comparisons of d' for geometric and d' for nonsense designs. Interestingly, bipolar probands, while generally performing on par with nonpsychiatric controls, also demonstrated less benefit (e.g., smaller effect sizes) from the geometric design type, as well as a greater loss of benefit following a delay relative to both nonpsychiatric controls and schizophrenia probands. On the CVLT, Recall style-by-group interaction failed to attain significance; as such, the hypothesis that schizophrenia probands would be more likely to use a less efficient learning strategy (e.g., serial rather than semantic) than nonpsychiatric controls or bipolar probands was not supported.

Expectations of diminished recognition discrimination in biological relatives of patients with schizophrenia compared to nonpsychiatric controls and biological relatives of patients with bipolar disorder were not supported in the current study in either nonverbal or verbal modalities. These findings may reflect a limitation of the study, namely that recognition may be a less sensitive measure of memory deficit than, for example, free recall. It also is possible that tests purporting to measure similar processes vary in their degree of sensitivity, resulting in variability across studies. In addition, some studies have noted that the largest effect sizes in memory deficits are associated with encoding rather than loss of learned material over time [43,80]. The tasks used in the current study did not allow for a direct comparison of nonverbal and verbal encoding processes. While meta-analytic reviews of cognitive deficits in first-degree biological relatives of patients with schizophrenia have found nonverbal memory deficits to be present, but at a lower magnitude than verbal memory deficits [21,29,43,81,82], there does appear to be some inconsistency across studies in the pattern of nonverbal memory deficits reported in the literature, as noted by Heinrichs and Zakzanis [29]. For example, a recent study by Skelley *et al.* [80] found a deficit in verbal memory, but nearly normal visual memory rates among siblings of patients with schizophrenia relative to controls and at odds with the schizophrenia probands in the study, who did demonstrate deficits in memory for both nonverbal and verbal material. As such, inconsistencies remain, and it appears the field would benefit from more in-depth exploration of nonverbal memory deficits in both schizophrenia probands and their biological relatives.

Females demonstrated higher performance on the verbal recognition memory task (CVLT). This finding held true across both sets of comparison groups, the group containing probands and nonpsychiatric controls, as well as the group containing relatives of probands and nonpsychiatric controls. Furthermore, females in the group containing proband relatives and nonpsychiatric controls were more likely than their male counterparts to utilize a semantic strategy for verbal recall. In the same group, there was a trend toward a greater discrepancy between immediate and delayed nonverbal recognition for males than females. The finding that females generally performed better than males on verbal recognition memory replicates an existing, well-established body of the literature demonstrating consistently higher performance in women compared to men across multiple tasks of verbal memory [83,84].

There is also evidence for gender differences in schizophrenia in cognitive domains beyond verbal memory [47,48,49], with women generally tending to perform better than men. This leads to discussion of another limitation of the current study, namely, that schizophrenia and bipolar proband groups contained a much higher proportion of males than females, which was not the case for the nonpsychiatric control group or either group of proband relatives. This disparity likely reflects the fact that the study took place at a Veterans Affairs medical center, where the patient population contains a disproportionately higher percentage of males. Although efforts were made to recruit participants from community and county clinics, the availability of female participants was limited. As such, limited conclusions can be drawn regarding gender differences in the proband comparison groups. Nevertheless, with this caveat in mind, it should be noted female probands with schizophrenia did perform better than their male counterparts on one condition of the nonverbal memory task, delayed recognition of geometric designs; in fact, the female probands with schizophrenia performed similarly to nonpsychiatric controls and bipolar probands in this condition (d' = 3.79 *vs.* 3.75 and 3.23, respectively). Future investigations would benefit from inclusion of a larger number of female probands to further explore gender effects.

There were several other limitations to this study. First, with an average age of 49.85 years, many of the relatives of patients with schizophrenia, although age-matched to the patients, may have been past the age of risk to develop the disorder, thus diluting the risk loading within this particular sample of relatives. Additionally, the nature of the computerized nonverbal learning task limited comparisons across nonverbal and verbal modalities to recognition memory. Compared to verbal memory tasks, nonverbal memory tasks tend to have more limited flexibility in what can be measured, and care must be taken to minimize options for use of language to assist with recall (e.g., objects that can be named) to avoid unintended confounds [77]. Moreover, free recall of nonverbal material tends to be dependent on motor and visuoconstructional skills (e.g., ability to draw). Future research directions might include younger and larger samples, as well as nonverbal and verbal tasks assessing a broader range of memory components.

## 5. Conclusions

Episodic memory deficits are one of the more prominent cognitive features in schizophrenia and have been demonstrated across nonverbal and verbal modalities. Similarly, meta-analytical studies have shown memory deficits in first-degree biological relatives of patients with schizophrenia, with effect sizes of small to moderate magnitude [43,81,82] and typically greater in verbal than nonverbal memory. Such findings may help clarify the role of cognitive deficits in identifying endophenotypes for the disorder. In the current study, probands with schizophrenia performed more poorly than nonpsychiatric controls and bipolar patients in both nonverbal and verbal recognition memory, consistent with the literature and confirming the specificity of these deficits to schizophrenia. Findings also suggested better performance by females with schizophrenia compared to their male counterparts, although this finding requires further exploration with larger female proband samples. Relatives did not demonstrate significant impairments in either nonverbal or verbal recognition memory, a finding that is inconsistent with meta-analytical studies, and it may reflect the heterogeneity of samples, small sample sizes, dilution of risk loading and/or limited sensitivity of the measure used. Improved understanding of the nature of episodic memory impairment in schizophrenia and its relationship to genetic predisposition for the disorder holds promise for helping identify neural substrates pertinent to etiology and recovery.
